# Phototherapy Combined with Carbon Nanomaterials (1D and 2D) and Their Applications in Cancer Therapy

**DOI:** 10.3390/ma13214830

**Published:** 2020-10-28

**Authors:** Prabhavathi Sundaram, Heidi Abrahamse

**Affiliations:** Laser Research Centre, Faculty of Health Sciences, University of Johannesburg, Johannesburg 2028, South Africa; prabhavathis@uj.ac.za

**Keywords:** cancer, carbon nanotubes, graphene, photodynamic therapy, photosensitizers, phototherapy

## Abstract

Carbon-based materials have attracted research interest worldwide due to their physical and chemical properties and wide surface area, rendering them excellent carrier molecules. They are widely used in biological applications like antimicrobial activity, cancer diagnosis, bio-imaging, targeting, drug delivery, biosensors, tissue engineering, dental care, and skin care. Carbon-based nanomaterials like carbon nanotubes and graphene have drawn more attention in the field of phototherapy due to their unique properties such as thermal conductivity, large surface area, and electrical properties. Phototherapy is a promising next-generation therapeutic modality for many modern medical conditions that include cancer diagnosis, targeting, and treatment. Phototherapy involves the major administration of photosensitizers (PSs), which absorb light sources and emit reactive oxygen species under cellular environments. Several types of nontoxic PSs are functionalized on carbon-based nanomaterials and have numerous advantages in cancer therapy. In this review, we discuss the potential role and combined effect of phototherapy and carbon nanomaterials, the mechanism and functionalization of PSs on nanomaterials, and their promising advantages in cancer therapy.

## 1. Introduction

Cancer is a deadly disease, where cells grow in an enormous amount and kill a huge population worldwide. Both developed and developing countries are largely affected and the death rate is high due to food habits, lack of exercise, genetic reasons, etc. Every year, the death rate increases in breast, lung, stomach, and liver cancers [1]. The exact cause of cancer is difficult to understand, and continuous research is underway to find cancer growths due to genetic disorders or external chemical compounds altering the gene by either addition or deletion, or overexpression of a gene, leading to the uncontrolled growth of cells [2]. Human papillomavirus, Helicobacter pylori, and hepatitis infections also lead to cancer incidence and progression [3]. Commercially available chemotherapy to treat cancer cells has numerous side effects such as high cost, low bioavailability, and poor targeting.

The ancient medical technique to cure diseases with the help of sunlight was followed in various countries like Egypt, China, and India, and this treatment was slowly forgotten over time due to modern medicine. In the 15th to mid-19th century, people used to cure skin diseases following sunlight therapy named heliotherapy [4]. The Atharvaveda Indians used plant extract and different Ayurvedic oil or seed extracts on the affected area for various diseases and then treated them with sunlight [5]. Nowadays, modern phototherapy is emerging faster in the medical field due to its efficient curing rate. The father of ultraviolet therapy, Niels Ryberg Finsen, designed a chemical ray lamp to treat Lupus vulgaris and succeeded [6]. Then, treatments like ultra vitalux lamps, fluorescent tubes [7], photochemotherapy, and psoralen and ultraviolet A (PUVA) slowly evolved in the 19th century [8]. During the 1980s, the term phototherapy was first named as extra corporeal photochemotherapy (extracorporeal photopheresis, ECP), and this was introduced to treat palliation of erythrodermic cutaneous T-cell lymphoma (CTCL) and was approved by the FDA in 1988 [9]. Thus, phototherapy attracted the research field and many treating methods were invented for various diseases like skin disease, cancer, dental care, and eye treatment.

## 2. Types of Phototherapy

### 2.1. Photobiomodulation (PBM)

Photobiomodulation (PBM) is an alternative medicine to treat various diseases by applying a low-level laser or low-power or light-emitting diodes with a limited wavelength level on the affected area of the body [10]. In the late 1960s, this technique was first introduced by Mester for hair growth in mice and potential medical applications [11]. PBM has been used to treat acute and chronic pain, wound healing [12], inflammatory disorders, dentistry [13], neurological disorders, head and neck cancer [14], Parkinson’s disease [15], colorectal cancer, carpel tunnel syndrome [16], and musculoskeletal syndrome [17]. PBM has both inhibitory and simulating effects when the light source is introduced into the cells [18,19]. The great opportunities of using blue and green light on stem cells in regenerative medicine [20], red light in spermatozoa motility [21], and visible light are being developed to visualize neuronal cells therapy [22].

The photobiomodulation effects on cell proliferation on most of the cells like fibroblasts, endothelial cells, keratinocytes, and lymphocytes are based on photo-stimulation of the mitochondrial signalling pathways, and increased production of growth factors by the regulation of transcription process [23,24,25]. Recent studies have focused on head and neck cancer therapy using PBM. The common side-effect of head and neck cancer was oral mucositis, and it was treated using PBM, achieving a positive response, which controlled the radiotherapy-induced oral mucositis [26].

### 2.2. Photodynamic Therapy (PDT)

**Figure 1:**
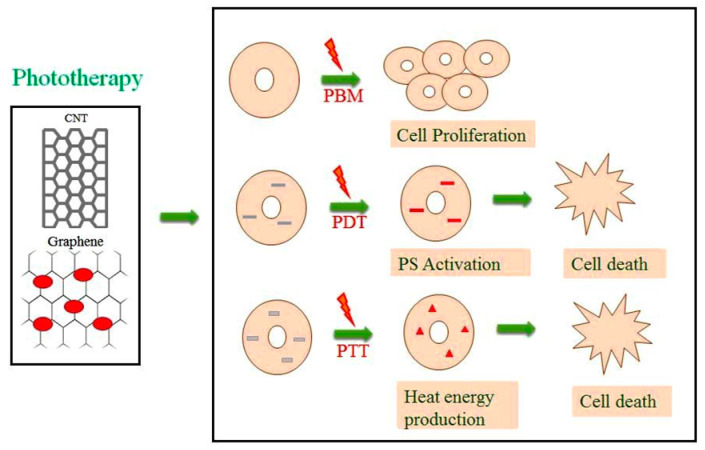
Mechanism action of phototherapy. CNT—carbon nanotubes, PS—photosensitizers, PBM—photobiomodulation mechanism of light source on cells leads to cell proliferation. PDT—photodynamic therapy mechanism of incorporation of PS into the cells with light source emission leading to PS molecule activation induces singlet oxygen production and cell death. PTT—photothermal therapy mechanism of addition of nanomaterials, which has thermal properties after the introduction of a light source, produces heat energy, leading to cell death.

Photodynamic therapy is an emerging medical treatment, and research is still ongoing in this century. PDT is a treatment modality that utilizes light of a specific wavelength to activate photosensitizers (PS) to destroy tumours. This works through a PS molecule or drug upon activation by a specific light to produce reactive oxygen species and that specifically kills the targeted tumours. [27,28]. PDT has a higher therapeutic efficacy and improves outcomes for cancer treatment in comparison to commercially available chemo and radiotherapy [29]. The first clinical PDT was tested by Dougherty and co-workers (1978), successfully treating cancer in preclinical models at Rosewell park cancer institute [30]. The PS drug has toxic effects on cancer cells when activated by the light source, but the drawback is that most of the PS molecule is water-insoluble in nature [31]. Therefore, to overcome the drawback, PS molecules have recently been coupled with different nanomaterials for significantly enhanced efficacy and tumour selectivity in cells using PDT [32,33]. The different mechanisms of phototherapy using carbon-based nanomaterials are shown in Figure 1.

### 2.3. Photothermal Therapy (PTT)

At present, photothermal therapy has attracted increasing attention in research due to the targeted ablation of cells using heat energy mainly on cancer. In PTT, the photosensitizer is subjected to a light source, and the PS molecule is activated for heat energy that leads to cell death [34]. When there is a rise in body temperature during the recovery of certain diseases, the elevated heat energy will slow down the multiplication of bacteria, viruses and pathogens, and cancer cells, which also lead to death in the heat environment. Recent research on PTT has been focusing on introducing nanoparticles into the cells, which would have the thermal effect and be activated by laser irradiation, mainly to treat cancer cells using particles like gold, silver, fullerene, carbon nanotubes, and graphene [35]. Photothermal therapy and commercially available chemotherapy combine to improve the therapeutic effects on the treatment of cancer, hence the attracting of researchers in recent years [36]. The types of phototherapy with commonly used wavelengths, lamps, mechanisms of action, and its applications are presented in Table 1.

## 3. Photosensitizers (PS)

Photosensitizers are light-sensitive molecules, which are available in natural and synthetic compounds. PSs are molecules widely used in PDT, and when the light source emitted at a particular wavelength transfers into the PS molecule, it reaches an excited state, and electron transfer will occur in the chemical reaction and produce cytotoxicity [50]. The photosensitizers are well-soluble in body tissues, and when coupled with the targeted nanomaterial, they easily reach the cells.

**Figure 2:**
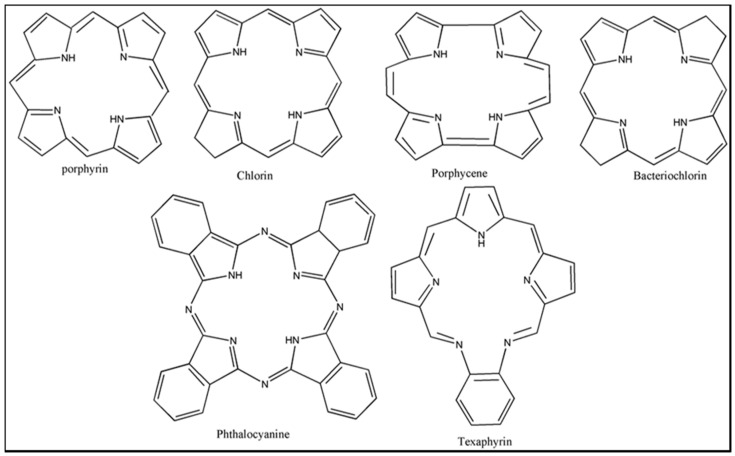
Examples of porphyrinoids: Porphyrin, Chlorin, Porphycene, Bacteriochlorin, Phthalocyanine, Texaphyrin.

**Figure 3:**
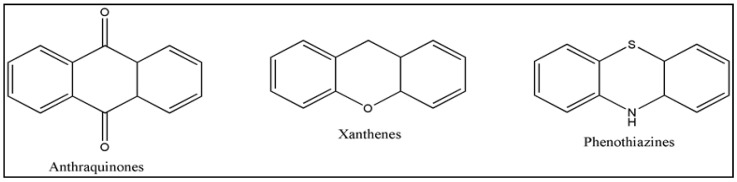
Examples of nonporphyrin: Anthraquinones, Xanthenes, Phenothiazines.

Photosensitizers are sensitive at a specific wavelength, where the minimum wavelength range between 400 and 600 nm prevents excessive sensitivity from sunlight, and the maximum absorption wavelength is between 600 and 800 nm; those above 800 nm will not provide excess oxygen production. It has minimal cytotoxicity in the dark, so when the nanoparticle carries the PS molecule, it will affect the normal cells until activating it [51,52,53]. In cancer therapy commonly, the used PS molecule is from tetrapyrrole compounds, which have a similar structure as protoporphyrin prosthetic group has in haemoglobin for their ease of efficacy to navigate inside living cells [54]. The photosensitizers are classified based on the evolution such as first, second, and third generation. The first-generation PS molecule was introduced by Oscar Raab in 1904, who explained to his professor Von Tappeiner that acridine dyes kill protozoa when irradiated [55]. A commercially available PS molecule was introduced in the 1970s by Dr. Thomas Dougherty and colleagues [56]. In the 1980s, second-generation PS molecule studies began and a few PS molecules were used in clinical trials for anticancer activity. The molecules were hematoporphyrin derivatives and synthetic photosensitizers [57]. These molecules deeply penetrate into the tissues, have a high yield for oxygen molecules in pure form, and get activated at wavelengths between 650 and 800 nm [58]. The third-generation PS molecules have a high affinity to the tumour cells; it will not affect the normal cells and can target the specific area in the body during PDT. More molecules are derived and synthesized to treat cancer cells combined with nanoparticles to improve the targeting of specific cells and to increase the bioavailability of PS [59]. Ormond et al., 2013 discussed the structures and activation energy of different PS molecules and the properties of photosensitizers, and its application in cancer is given in Table 2 [60]. In the use of cancer treatment, most of the PS molecules have porphyrinoid and nonporphyrin dyes, and the modified form of this group is used in cancer cells. Some examples of porphyrinoid and nonporphyrin PS are shown in Figure 2 and Figure 3.

## 4. Carbon Nanomaterials

The carbon nanomaterials are classified as zero-dimensional (0D)-structure fullerene, one-dimensional (1D) carbon nanotubes (CNT) and two-dimensional (2D) graphene nano-sized molecules. They are widely used in electronic and electrical fields, biosensors, medical treatments, environmental, etc. [78]. This nanomaterial has unique structures and physiochemical characteristics in the biomedical field to avoid current chemotherapeutic toxicity and to provide new effective therapies. In the drug delivery system, CNTs and graphene play significant roles compared to other nanomaterials due to the large surface area, so their loading efficiency of drugs or biomolecules or PS are high [96].

### 4.1. One Dimensional Carbon Nanotubes (CNT)

Carbon nanotubes are like rolled-up graphene sheets in a hollow cylindrical shape where both the ends are opened, and carbon atoms are exclusively arranged like benzene rings. The structural representation of CNT is armchair, zigzag, and chiral, with allotropic forms of both sp^2^ planar and sp^3^ cubic [97]. When the single sheet is rolled up and forms a tube, it is named a single-walled carbon nanotube (SWCNT) sized between 1 and 3 nm in diameter, with a length of several micrometres. Multiple sheets that roll up and form a tube are named multi-walled carbon nanotubes (MWCNTs) sized between 1 and 3 nm inner diameter, 2 and 100 nm outer diameter, and a length varying from 0.2 to several micrometres [98]. Synthesis of CNTs is mainly in three processes, namely the discharge, chemical vapor deposition (CVD), and laser ablation techniques. Some natural techniques are available but have no proper yield or standardized method [99]. The purity of the CNT is achieved by the acid reflux method, air oxidation, and surfactant-based sonication to remove the extra metals, for when it goes to biological applications as purity is very important [100]. Carbon nanotubes play a unique role as nanocarriers to deliver drugs, polymers, photosensitizers, and specific ligands to target siRNA and DNA. In cancer therapy, conventional treatment like surgery, chemotherapy or radiotherapy has numerous side effects and does not completely get rid of the disease, due to poor targeting, bioavailability, and damaging organs [101]. Now, scientists are focusing on combination therapy like nanomaterials with phototherapy for effective and target cancer treatment. The photosensitizers are coupled with CNTs to increase the solubility, PS bioavailability, and targeting only the cancer cells [102]. The photothermal activity of the CNTs activated at the wavelength of 808 nm and PSs-coupled CNTs are used to treat cancer cells by photodynamic effects [103].

### 4.2. Two-Dimensional Graphene

Graphene nanomaterials have attracted more attention in several fields due to the presence of more functional groups, wide surface area, and biocompatibility. Geim and Nosovelov were the first to separate a single graphene sheet layer from graphite material in 2004, where they followed the mechanical cleaving technique for isolation from the graphite crystal [41]. It has an sp^2^-hybridized honeycomb structure with a two-dimensional carbon lattice, which has unique electronic properties. It has a relatively high Young’s modulus, faster electron mobility, and high electric and thermal conductivity. Graphene is widely used in the form of graphene oxide (GO), carboxyl graphene (GCOOH), and reduced graphene oxide (rGO) [104]. Graphene oxide synthesis from graphite gives a high yield of production and cost-effectiveness. Graphene has a wide range of applications in the electronic and electrical, biomedical, environmental, and nanotechnology fields. Graphene is a good nanovehicle to carry more drugs or PS molecules due to the wide surface area. Graphene produces photothermal activity when introduced at a wavelength of 808 nm [105]. The functionalized graphene nanomaterial surface bonds will be broken due to high-temperature vibration and the carrier molecules released inside the cells, which helps to target the cells and act as a good nanocarrier in biological applications [106].

## 5. Mechanism and Loading of PS on Nanomaterials

### 5.1. Physical Loading of PS

In the drug delivery system, nanoparticles will be loaded with chemotherapeutic drugs, biomolecules, PS, etc. and the loading of the molecule will be based on the physical and chemical characteristics. Physical loading, also called noncovalent bonding, is a process that has a different mechanism like hydrophobic interaction, van der Waal’s force, and π–π stacking. 1D and 2D particles without any functional groups are hydrophobic in nature, and when interacting with a nonpolar PS molecule, both adsorb on the surface, but it is not strong enough to enter into the body to treat cancer cells [107]. The van der Waal’s force involves dipole interaction between the nanoparticle and PS molecule by the intermolecular force, as if the PS aggregated on the surface of the nanoparticle. Both mechanisms are not well preferred in the drug delivery system, due to poor stability, loading efficiency and release moiety [108]. π–π stacking is another physical adsorption method to load PS on the nanoparticle. Both 1D and 2D have an aromatic ring carbon particle, and when the PS molecule has a similar structure, both stack on the surface, and noncovalent bonding interaction takes place by π bonds. Hence, this mechanism is named π–π stacking. The long-chain polymers or peptides will fold on the nanoparticles by π–π stacking to load drugs [109]. The advantages of noncovalent bonding is that there is no structural damage of the drugs; the properties of the nanoparticle will remain the same. Examples of π–π-stacked molecules are proteins and DNA [110], si-RNA [111], polyacrylic acid, and pyrene [112]. Most of the PS molecules like m-tetrahydroxyl phenylchlorin (mTHPC), zinc phthalocyanine, zinc monoamino phthalocyanine, and Chlorin e6 are coupled noncovalently by π–π stacking to treat various cancer cells using phototherapy, and PDT and PTT are commonly used in cancer cells [113].

**Figure 4:**
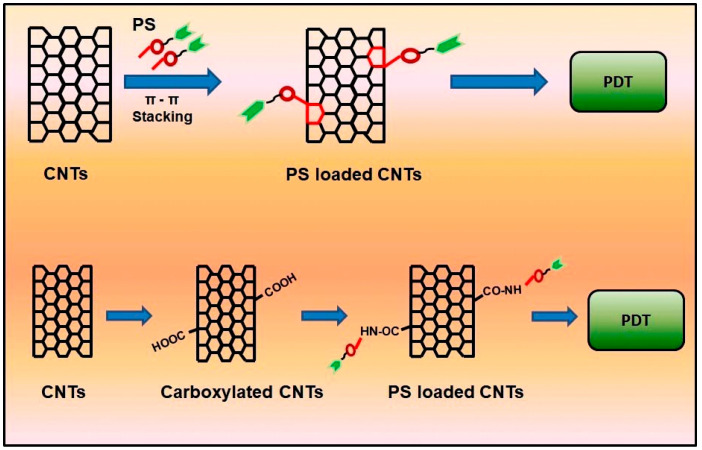
Loading mechanism of PS on nanomaterials. The attachment of the PS molecule on aromatic rings of CNTs by π–π stacking, and the carboxyl group into amide formation of PS on the walls of CNTs, followed by the molecules being subjected to PDT. (CNTs—carbon nanotubes, PS—photosensitizers, PDT—photodynamic therapy, COOH—carboxyl group, CONH—amide group).

In recent research, the graphene oxide composite was prepared for phototheranostic application purposes, and some of the composites were discussed. Photosensitizer chlorin e6 is coupled noncovalently by the ultrasonication technique for photodynamic and photothermal effects, and GO is coupled with PEG-coupled gold nanostars. This composite has a combined effect of PDT, PTT, and photoimaging both in vitro and in vivo to treat breast cancer with successful results [114]. Single-walled carbon nanotubes were coupled with an encapsulated albumin chlorin e6 PS molecule by the ultra-homogenization technique for PTT, having an effect on squamous cell carcinoma (SCC 7) cell lines for in vitro and in vivo studies on the BALB/c (Bagg and Albino laboratory-bred mouse strain) nude female mice model [115]. The multiwalled carbon nanotubes were utilized for both the PDT and PTT effect by coupling foscan^®^ (mTHPC) π–π stacking using a continuous stirring technique for 3 days, and the cell death mechanism occurred when irradiated at 650 and 880 nm on human ovarian cancer SKOV 3 cell lines [102]. The loading mechanism of PS on nanomaterials is depicted in Figure 4.

### 5.2. Chemical Loading of PS

The covalent functionalization is like a defect in the sidewalls of the nanomaterial, where there will be an addition of different functional groups [116]. The main aim of the covalent functionalization is to avoid the change in physical properties like solubility, purity, and sp^2^ and sp^3^ hybridization of carbon molecules. Those properties of carbon have a wide range of applications in the field of medical science and nanotechnology [117,118,119,120]. The functional groups are added to the CNTs like the carboxyl group, hydroxyl, amine, fluoride, and disulphide bonds [121]. The carboxyl group of graphene oxide and the amine group of polyethylene glycol (PEG) are coupled using the EDC-NHS method to act as a high loading nanocarrier [122]. Due to the solubilizing nature and biocompatibility of the functionalized carbon nanomaterials, it is opted to add different polymeric chains, proteins, DNA, and drugs. In a recent study, polyethyleneimine-functionalized SWCNTs are tested on melanoma cells in vitro and in vivo using PDT. The single-walled carbon nanotubes covalently functionalized composite showed excellent photocytotoxic action against cancer cells, and the activity of the composite was based on the functionalization method [123].

## 6. Application of Phototherapy Using CNT and Graphene on Cancer Therapy

Various research works are ongoing using the photo effect to treat new diseases to overcome the side-effects of present chemotherapy mainly on cancer. Using CNT and graphene nanomaterials for PDT, PTT, and photo imaging by coupling with different PS targets cancer cells and fluorescent molecules. The different nanoparticles with photosensitizers and their applications are listed in Table 3.

## 7. Conclusions and Future Perspectives

Carbon-based nanomaterials graphene and carbon nanotubes play a vital role as nanocarriers at present, and more research articles have been published as they are a good carrier to treat cancer cells both in vivo and in vivo. The unique structure of CNT and graphene will increase the loading efficiency of PS molecules, the biocompatibility, bioavailability, and thermal effect. The phototherapy using carbon nanomaterials to treat cancer is a new approach and is effective with reduced side effects. The loading mechanism of photosensitizers is clearly studied based on the structure and functional group present in the PS molecule. Most of the PS molecules have a benzene ring in their structure that will attach on the nanomaterials by π–π stacking noncovalent functionalization. The chemical functionalization with the PS molecule has very few research works being done to treat cancer cells. The surface functionalization will improve the targeting of the therapeutic efficiency of the photosensitizers for the treatment of cancer in PDT. More research work is needed on this perspective of surface modifications to develop novel targeted carbon nanomaterials for the treatment of cancer. Furthermore, more in vitro, in vivo, and clinical trials are recommended to unlock the medicinal applications of carbon-based nanomaterials for the treatment of cancer. The carbon nanotube and graphene-based nanocomposite with the PS molecule are used to treat various cancer cells, and the mechanism of loading with target molecules is detailed in this review. Emerging phototherapy combined with carbon nanomaterials will be an alternative treatment for cancer with fewer side effects.

## Figures and Tables

**Table 1:** Types of phototherapy.

	Wavelengths	Lamps	Mechanism of Action	Applications	Reference
**PBM**	500–1100 nm	Neodymium:yttrium-aluminium-garnet (Nd:YAG) laser, helium-neon laser (He-Ne), Diode laser.	The low-level light source at a particular wavelength applied into the cells will stimulate or enhance the cells.	Rheumatoid arthritis, osteoarthritis, wound healing, low back pain.	[37,38,39,40]
**PDT**	400–800 nm	NIR lasers, diode lasers, UV lights.	The PS molecule in the ground state; when the molecule activated by the light source reaches the excited the state, it converts to the triplet state by electron spinning. The triplet state interacts with the surrounding oxygen molecule and produces ROS through type I and type II reaction	Antimicrobial, fungal, viral activity, acne vulgaris, malignant tumour (lung, skin, head and neck, prostate cancer), wound healing.	[41,42,43,44,45,46]
**PTT**	800–980 nm	NIR lasers, UV lights.	The particle (PS or nanoparticles) will be activated by the light source and produce heat energy.	Prostate cancer, melanoma skin cancer, Alzheimer’s disease.	[47,48,49]

PBM—photobiomodulation, PDT—photodynamic therapy, PTT—photothermal therapy, NIR—near-infrared, PS—photosensitizers, UV—ultraviolet.

**Table 2:** Properties of photosensitizers and its application in cancer.

PSs	Structure	Λ(nm) and (Εmax)	Application
**HpD**	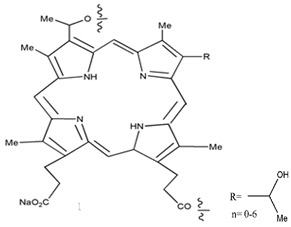	630 nm and 3000 (M^−1^ cm^−1^)	Brain, Lung cancer [60,61]
**Photofrin**	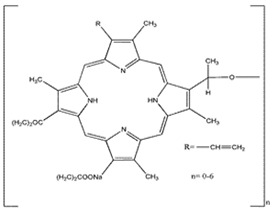	630 nm and 3000 (M^−1^ cm^−1^)	Bladder, Lung, Oesophagus cancer [62,63]
**5-Aminole-vulinic acid**	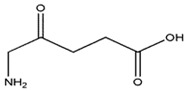	632 nm and 5000 (M^−1^ cm^−1^)	Cancer diagnosis [64,65,66]
**Methyl aminolevulinate**	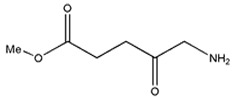	632 nm and 5000 (M^−1^ cm^−1^)	Nonmelanoma cancer, Basal cell carcinoma [67,68,69]
**Hexaminolevulinate**	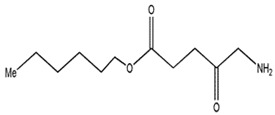	632 nm and 5000 (M^−1^ cm^−1^)	Bladder cancer diagnosis [70,71,72]
**Lu-Tex**	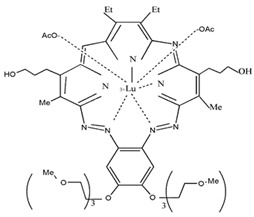	732 nm and 42,000 (M^−1^ cm^−1^)	Prostate cancer, Breast cancer, Cervical cancer [73,74,75]
**Meta-tetra(hy-droxyphe-nyl)por-phyrin**	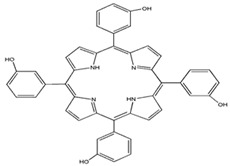	648 nm	Basal cell Carcinoma, Head and neck cancer and Skin cancer [76,77,78]
**5,10,15,20-tetrakis(4-sulfanato-phenyl)-21H,23H-porphyrin**	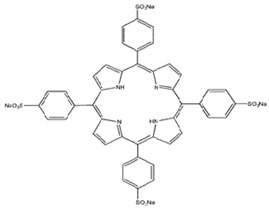	645 nm	Basal cell Carcinoma [27,63,79,80]
**N-aspartyl chlorin e6**	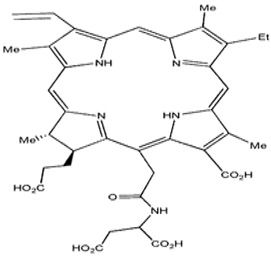	664 nm and 40,000 (M^−1^ cm^−1^)	Lung cancer [81,82]
**2-(1-hexylo-xyethyl)-2-devinyl Pyropheo-phorbide**	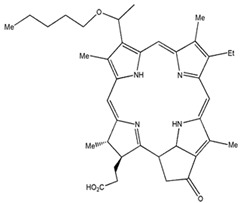	665 nm and 47,000 (M^−1^ cm^−1^)	Oesophageal cancer, Basal cell carcinoma, Lung cancer [83,84]
**Phenothia-zines**	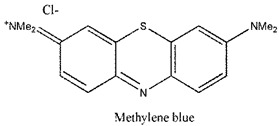	666 nm and 82,000 (M^−1^ cm^−1^)	Basal cell Carcinoma, Kaposi’s sarcoma, Cervical cancer [85,86]
**Padoporfin**	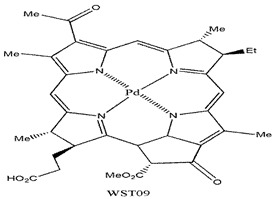	763 nm and 88,000 (M^−1^ cm^−1^)	Prostate cancer [87]
**Aluminium phthalo-cyanine-tetrasulfo-nate**	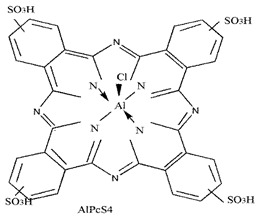	676 nm and 200,000 (M^−1^ cm^−1^)	Stomach, Skin, Oral, Breast cancer [88]
**Curcumi-noids**	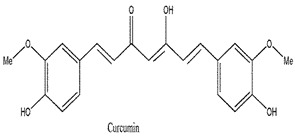	420 nm and 55,000 (M^−1^ cm^−1^)	Breast, Skin cancer [89,90]
**Xanthenes**	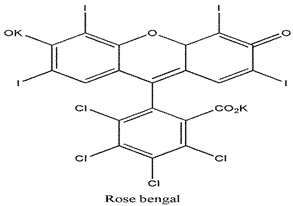	549 nm and 100,000 (M^−1^ cm^−1^)	Breast carcinoma and Metastatic melanoma [91]
**4,5-Dibromorhodamine methyl ester**	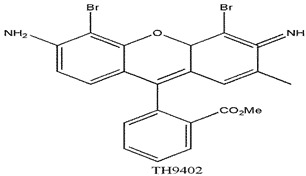	514 nm and 100,000 (M^−1^ cm^−1^)	Breast cancer [92]
**Anthraquinones**	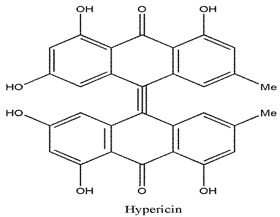	590 nm and 44,000 (M^−1^ cm^−1^)	Squamous cell carcinoma and Basal cell carcinoma [93,94]
**Meta-tetra(hydroxyphenyl)chlorin**	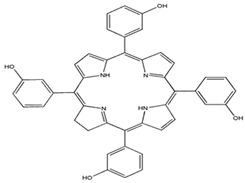	652 nm and 35,000 (M^−1^ cm^−1^)	Breast, Prostate, Pancreatic cancer, Head and neck cancer [77,80,95]

**Table 3:** Various applications of phototherapy using 1D and 2D carbon nanomaterials on cancer.

Nanoparticle	Photosensitizer	Bonding	Photo Therapy	Applications	Targeted	References
SWCNTs	-	Covalent (Carboxyl-amine cross-linking)	PTT	Glioblastoma cells	Anti CD133	[124]
SWCNTs	SWCNT-PEI SWCNT-PVPk30	Covalent (cationic polymerization) and noncovalent (physical attachment)	PDT	Mus musculus skin melanoma cells (B16-F10 cells)	-	[102]
Metallic-SWCNT Semiconducting-SWCNT	-	-	PDT PTT	Lung cancer cells (NCI-H460)	-	[125]
MWCNT	m-tetrahydroxylphenylcholrin	Non-covalent (physical attachment)	PDT PTT	Ovarian cancer cells (SKOV3)	-	[103]
SWCNT (Evans blue and albumin)	Chlorin e6	Non-covalent (physical attachment)	PDT/PTT	Mouse squamous cancer cells (SCC-7) and In vivo	-	[116]
SWCNT	Zinc phthalocyanine	Covalent (carboxyl-amine cross-linking) and non-covalent (physical attachment)	PDT	Breast cancer cells (MCF 7)	Spermine	[126]
SWCNT (chitosan)	Chlorin e6	Non-covalent (π-π interaction)	PDT	HeLa cancer cells	-	[127]
SWCNT, GO, Fullerene	-	Covalent (Carboxyl-amine cross linking)	PTT	Breast cancer cells (MCF 7) and In vivo	Hyaluronic acid	[128]
SWCNT	Zinc monoamino phthalocyanine	Covalent (Carboxyl-amine cross linking) and non- covalent (π-π interaction)	PDT/PTT	Melanoma cells (A375)	Folic acid	[129]
SWCNT (Docetaxel NGR peptide)	-	Non-covalent (physical attachment)	PTT	Human prostate cancer (PC3) and in vivo	-	[130]
CNT Graphene sheet	Hydroxyapatite	Non-Covalent (physical attachment)	PTT	-	-	[131]
Graphene oxide-PEG-DOX	-	Covalent (Carboxyl-amine cross-linking)	PTT	Murine mammary cancer cells (EMT6) and in vivo	-	[123]
Graphene oxide-gold nanorods-Doxorubicin	-	Noncovalent (physical attachment)	PTT	Lung cancer cells (A549)	-	[132]
Reduced graphene oxide	-	Noncovalent (physical attachment)	PTT	Human breast cancer (MCF 7)	Hyaluronic acid	[133]
Graphene oxide Palladium	-	Covalent (Carboxyl-amine cross-linking)	PDT PTT	Human prostate cancer (PC3)	-	[134]
Graphene	-	-	PDT PTT	Cervical cancer cells (HeLa)	-	[135]
Graphene oxide-PEG	-	Covalent (Carboxyl-amine cross-linking)	PDT PTT	Melanoma cells (B16F0)	Folate	[106]
Graphene oxide (Quantum dots)	TRITC	Covalent (Carboxyl-amine cross-linking) Noncovalent (physical attachments)	PDT	Mouse mammary tumour cells (4T1)	Upconversion nanoparticle (UCNP)	[136]
Graphene	-	Covalent (Carboxyl-amine cross-linking)	PTT	Skin cancer (in vivo)	Hyaluronic acid	[137]
Graphene oxide	Chlorin e6	Noncovalent (π-π interaction)	PDT/ PTT	Mouse breast mammary carcinoma cells (EMT6)	-	[115]
Graphene oxide-PEG-Folic acid	-	Covalent (Carboxyl-amine cross-linking)	PTT	Human breast cancer (MCF 7 and MDA-MB-231)	Folate	[138]
SWCNTs	Chlorin e6	Non-covalent (π-π interaction)	PDT	Human colon cancer cells (Caco 2)	Hyaluronic acid	[139]

## Abbreviations

0D	Zero dimensions
1D	One dimension
2D	Two dimensions
CD	Compact disk
CNT	Carbon nanotubes
COOH	Carboxyl group
CTCL	Cutaneous T-cell lymphoma
CVD	Chemical vapor deposition
DVD	Digital video disk
ECP	Extracorporeal photopheresis
EDC	Ethyl(dimethylaminopropyl) carbodiimide
FDA	Food and drug administration
GCOOH	Carboxylated graphene
GO	Graphene oxide
He-Ne	Helium and neon
HpD	Haematoporphyrin Derivative
IEC	International Engineering Consortium
LLLT	Low-level laser therapy
M^−1^ cm^−1^	Per moles per centimetre
mTHPC	m-tetra hydroxyl phenylchlorin
MWCNT	Multi-walled carbon nanotubes
Nd:YAG	Neodymium-doped yttrium aluminium garnet
NHS	**N** -Hydroxysuccinimide
NIR	Near-infrared
Nm	Nanometre
OH	Hydroxyl group
PBM	Photobiomodulation
PDT	Photodynamic therapy
PEG	Polyethylene glycol
PEI	Polyetherimide
PS	Photosensitizers
PTT	Photothermal therapy
PUVA	Psoralens and ultraviolet A
PVPk30	Polyvinylpyrrolidone
rGO	Reduced graphene oxide
ROS	Reactive oxygen species
siRNA	Small interfering ribonucleic acid
DNA	Double-stranded nucleic acid
SWCNT	Single-walled carbon nanotubes
TRITC	Tetramethylrhodamine
UCNP	Upconverting nanoparticles
UVA	Ultraviolet A
UVB	Ultraviolet B
εmax	Molar extinction coefficient
